# Digital Interventions for Managing Medication and Health Care Service Delivery in West Africa: Systematic Review

**DOI:** 10.2196/44294

**Published:** 2024-10-09

**Authors:** Emmanuel Oluwatosin Oluokun, Festus Fatai Adedoyin, Huseyin Dogan, Nan Jiang

**Affiliations:** 1 Department of Computing and Informatics Faculty of Science and Technology Bournemouth University Poole United Kingdom

**Keywords:** digital interventions, medications delivery, phone-based intervention, tele and e-based intervention, West Africa, management, technology, intervention, medication, tool, smartphone

## Abstract

**Background:**

As a result of the recent advancements in technology, the incorporation of digital interventions into the health care system has gained a lot of attention and adoption globally. However, these interventions have not been fully adopted, thereby limiting their impact on health care delivery in West Africa.

**Objective:**

This review primarily aims at evaluating the current digital interventions for medication and health care delivery in West Africa. Its secondary aim is to assess the impacts of digital interventions in managing medication and health care service delivery with the intent of providing vital recommendations that would contribute to an excellent adoption of digital intervention tools in the health care space in West Africa.

**Methods:**

In line with PRISMA (Preferred Reporting Items for Systematic Reviews and Meta-Analyses), a comprehensive search through various databases yielded 529 results. After a rigorous screening, 29 articles that provided information on 3 broad digital health intervention tools were found eligible for this review.

**Results:**

Out of 29 studies, 16 (55%) studies examined phone-based interventions, 9 (31%) studies focused on tele- and e-based interventions, and 4 (14%) studies evaluated digital interventions. These interventions were used for diverse purposes, some of which are monitoring adverse drug reactions, general health, sexual and reproductive health, and training of health care practitioners. The phone-based intervention appears to be the most known and impactful of all the interventions, followed by tele- and e-based, while digital interventions were scarcely used.

**Conclusions:**

Digital interventions have had a considerable level of impact on medication and health care delivery across West Africa. However, the overall impact is limited. Therefore, strategies must be developed to address the challenges limiting the use of digital intervention tools so that these tools can be fully incorporated into the health care space in West Africa.

## Introduction

### Background

In the last few decades, information technology has witnessed huge transitions [1]. These have facilitated the incorporation of programs and electronic devices using digital technology (digital intervention) for medication delivery and the health care system globally [2,3]. Recently, a group of researchers from the University of Michigan established that mobile health apps permit users to be more knowledgeable about their health, track behavioral changes in health, and obtain digital health assistance through a smartphone or tablet [4]. In the same vein, experts in the health care system have been reported to use mobile devices to connect with patients for consultation, monitoring, management, and making clinical decisions [5]. Furthermore, the use of mobile devices has been established to advance communication between health care experts and their colleagues [6].

Over the years, several digital intervention tools such as telephone-based interventions, web-based interventions, and mobile apps have been used for supporting smoking cessation, vaccine rates, disease management (type 2 diabetes, hypertension, cancer, etc), and medication delivery [7-13]. Studies have also revealed that digital intervention resulted in health benefits [14]. Hence, these previous studies have therefore provided strong evidence that depicts how digital interventions have been employed in medication delivery and the health care system [15].

There has been a plethora of reviews on the integration of digital interventions into the health care system. Recently, Giravi et al [16] conducted a mini review on adjunct digital therapies for pain management, while Ibrahim et al [17] also conducted a systematic review of studies on digital health for quality health care. The outcome of these reviews revealed that digital interventions have had positive impacts on health. However, most of these reviews have focused on the impacts of integrating digital interventions into the health care system outside Africa, while a few are within Africa. To date, no study has systematically reviewed the digital interventions for medication delivery in West Africa.

In most African countries, health care systems are typically in deplorable situations with severely meager health outcomes [18]. Although, Africa makes up a little above a tenth of the global population, its disease burden stands at 24%, while Sub-Saharan Africa has access to less than 1% of global health expenditure [19]. Sub-Saharan Africa appears to be the worst region in the world, with most countries below the World Health Organization standards for basic health care [20]. Unfortunately, West Africa, being part of sub-Saharan Africa, tops the list of least developed countries in Africa, with a total of 12 countries enlisted [21]. Thus, the need to focus on West Africa. Given these potentials of digital intervention in health care delivery and the peculiarity of West Africa, this study was consequently designed to achieve a systematic review of the digital interventions used for medication and health care delivery in West Africa to assess its impact and provide recommendations that would improve its overall integration into the health care space.

### Research Questions

This study aimed to evaluate the current digital interventions for medication and health care delivery in West Africa and also assess their overall impact in the health care space. Consequently, this review paper asks pertinent questions: What digital interventions are currently used for medication and health care delivery in West Africa? How well have these interventions been able to impact the health care system among West Africans and what are the challenges limiting its widespread adoption?

## Methods

### Overview

This systematic review uses PRISMA (Preferred Reporting Items for Systematic Reviews and Meta-Analyses) guidelines [22], and information on search strategy, terminologies, inclusion and exclusion criteria, study selection, and data extraction is given in detail in subsequent sections. However, the review is not registered on the International Prospective Register of Systematic Reviews. This is because, while International Prospective Register of Systematic Reviews accepts systematic review protocols assessing interventions (including qualitative and individual participant data reviews), the nature of intervention for this review is mainly digital interventions in terms of technology and not medical or public health interventions. This is a potential limitation to this review, and future reviews could consider a deeper review and balance of both digital and non-digital (medical and public health) interventions.

### Search Strategy

A comprehensive search through databases and various repositories was carried out using the PRISMA model for literature reviews [22]. Google Scholar, CINAHL, Web of Science, HubMed, BioMed, PubMed, Ovid, African Journals Online, and Scopus databases were searched to retrieve relevant information. Given that French and English are the two official languages in West Africa, our search encompassed databases in both languages.

### Terminologies

The terminologies used in the search comprised “digital intervention,” “digital health,” “digital technology,” “mHealth,” “eHealth,” “medication delivery,” and “West Africa.” The keywords imputed into the databases included; (“Digital Intervention” AND Nigeria OR “West Africa” AND “Medication management” OR “medication delivery” OR “health care service delivery” OR “healthcare service delivery” AND “Health management” OR “disease management”). The same search approach was followed for all other countries in West Africa, which include Benin, Burkina Faso, Cape Verde, Côte D'Ivoire, Gambia, Ghana, Guinea, Guinea-Bissau, Liberia, Mali, Mauritania, Niger, Nigeria, Senegal, Sierra Leone, and Togo with findings from the reviewed studies summarized in Multimedia Appendix 1.

### Inclusion and Exclusion Criteria

In line with the recommended guidelines for conducting a systematic review [22], certain conditions were fulfilled to conclude the inclusion and exclusion criteria for the research items used (Table 1).

**Table 1:** Summary of inclusion and exclusion criteria.

Inclusion criteria	Exclusion criteria
**Article type**	
Reviewed journals and articles Published materials, ie, case reports and case series on digital health intervention and medication delivery or general health care system between 2000 and 2022 Studies showing significant health improvement and promoted well-being	Editorials, letters, and protocol papers Conference abstracts Studies showing no remarkable improvement in health Studies without a control group Publications on the subject matter about countries outside West Africa
**Language**	
Studies published in English Studies published in French	Studies not published in English Studies published in French without comprehensive English translations

### Data Extraction

Data extraction was independently carried out by 2 reviewers. The following reports were drawn: (1) authors, year, country, and reference; (2) journal; (3) type of health issue and health technology; (4) research approach/method; (5) main findings; and (6) critical appraisal of the strength and limitation of each research item.

### Selection of Theme

**Figure 1:**
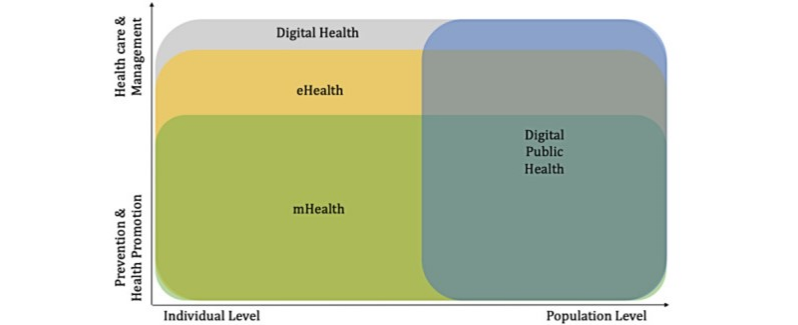
Core themes in the digitization of the health care system; mHealth, eHealth, digital health, and digital public health [24-32]. mHealth: mobile health.

The digitization of health care systems is referred to by terms such as eHealth, mHealth (mobile health), or digital health [23]. Understanding the significant variability of how these concepts interact with one another and their suitability in the digital health space is crucial given the diversity of terms and definitions used in the field of digital health. As a result, the themes used in this review were designed in line with these terminologies while we critically studied the articles so as to assign the various interventions under these themes, as seen in Figure 1 [24-32].

## Results

### Overview

The papers reviewed examined different digital interventions, including mobile, digital, and electronic technologies used in the health care system. Diverse research approaches were adopted by each of the papers, ranging from qualitative to quantitative, while some used assorted research methods. The terms used in the study include “digital interventions,” “digital technology,” “mobile health,” “eHealth,” and “Telehealth.”

### Study Selection and Paper Quality Assessment

**Figure 2:**
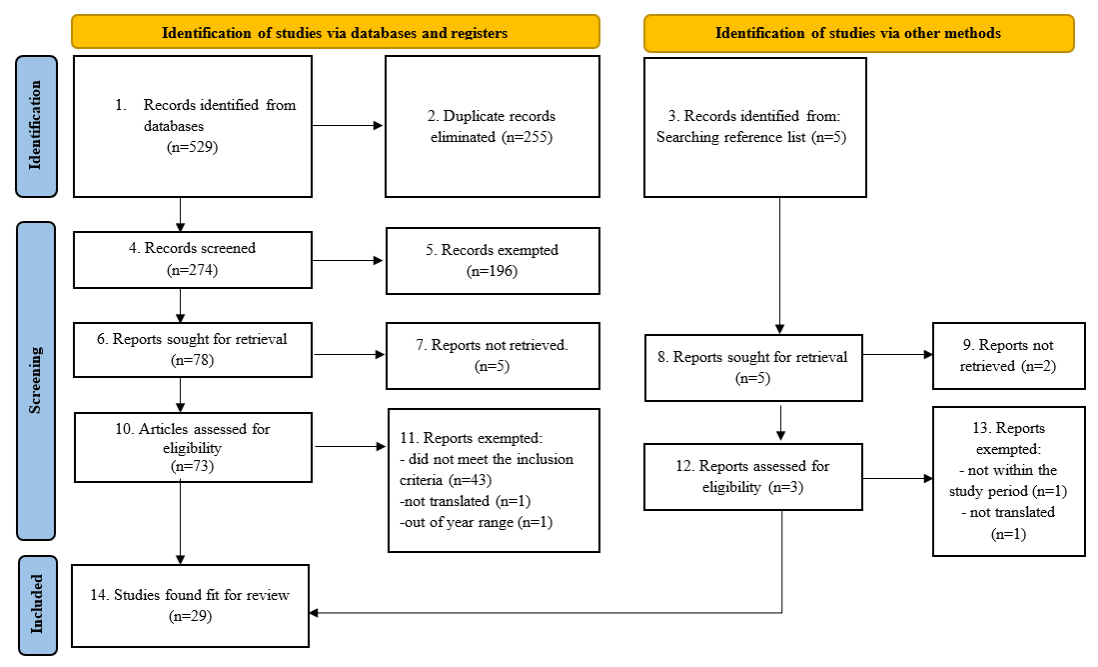
PRISMA (Preferred Reporting Items for Systematic Reviews and Meta-Analyses) flowchart of the study selection and inclusion process. Flow diagram retrieved from Page et al [22].

After a rigorous screening exercise in line with PRISMA guidelines (Multimedia Appendix 2), the flow diagram shown in Figure 2 presents a stepwise approach to the procedure used in study selection. From the search strategy, 529 records were retrieved from our various sources, out of which 255 duplicates were eliminated. A thorough screening was carried out on 274 records that had potential relevance via a careful examination of the abstracts and titles. Two members of the review team applied the exclusion criteria and found 78 studies eligible. At this point, an independent review of the preliminary result was carried out by 2 other members of the review team, of which only 29 (40%) were included in this review. Meanwhile, 5 articles were obtained by searching the citations of the 29 papers earlier selected. Out of the 5 articles obtained, only one additional article was selected after subjecting them to the selection criteria. Disagreement during screening between 2 reviewers was fixed via deliberation among the entire review team. Although a thorough check of each paper included in the review has been conducted following existing quality checks such as CASP (Critical Appraisal Skills Programme) checklists, other formal checks such as Joanna Briggs Institute’s critical appraisal tools can also be beneficial.

### Location of Studies Included

**Figure 3:**
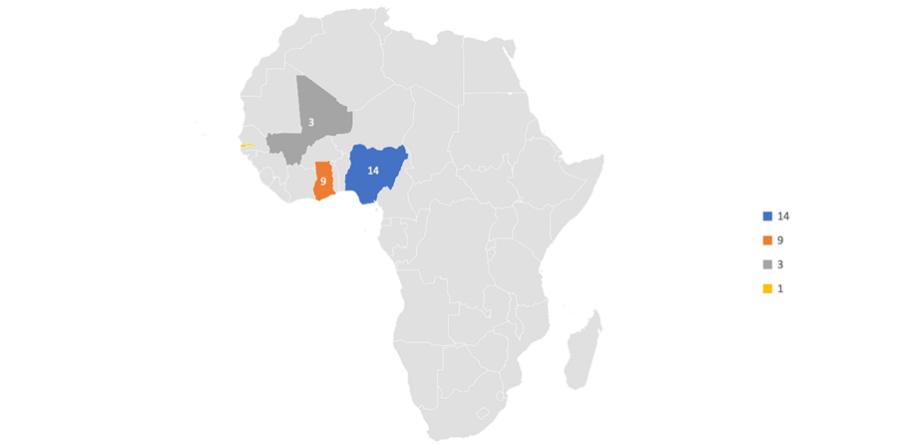
Papers reviewed per country. Source: Authors’ compilation.

Figure 3 shows that there were 14 papers from Nigeria, 9 from Ghana, 3 from Mali, 1 from Gambia, and 3 from other African countries, while there was no publication from other West African countries.

### Characteristics of Interventions and Sources of Evidence

This review had 16 articles evaluating phone-based interventions, while 9 articles examined tele- and e-based interventions and 5 focused on digital interventions. These are presented pictorially in Multimedia Appendix 3. We compressed the major findings from the study into 3 themes based on the interventions identified. These themes capture the key digital health interventions used in the health care system in West Africa. Of all the papers reviewed, mobile phone caller tunes, SMS text messages, phone calls, mobile apps, slide projectors, video training, data digitization, amplifier systems, educational digital storytelling, SatCom (satellite communications), and 3G mobile networks are the frequently occurring themes. Nevertheless, we distinguished the definite themes that characterize the core findings of each paper with a tick (Table 2): phone-based interventions, tele- and e-based interventions and digital interventions.

**Table 2:** Themes coined from the papers under review.

References	Theme 1: Phone-based interventions	Theme 2: Tele- and e-based interventions	Theme 3: Digital interventions
[33]	✓		
[34]	✓		
[35]			✓
[36]	✓		
[37]	✓		
[38]		✓	
[39]			✓
[40]		✓	
[41]		✓	
[42]		✓	
[43]	✓		
[44]	✓		
[45]	✓		
[46]			✓
[47]	✓		
[48]	✓		
[49]	✓		
[50]	✓		
[51]	✓		
[52]	✓		
[53]			✓
[54]	✓		
[55]	✓		
[56]	✓		
[57]			✓
[58]		✓	
[59]		✓	
[60]		✓	
[61]		✓	
[62]		✓	

### Theme 1: Phone-Based Interventions

It was found that toll-free telephone lines may be used to improve pharmacovigilance and follow-up on medication response in an environment with a paucity of funds [50]. In the same vein, Appiah et al [51] and Kukula et al [52] also supported that telephone follow-up and mobile caller tunes, respectively, should be used by patients for reporting ADRs. In line with these studies, the National Prescribing Service in Australia has successfully used a helpline to monitor adverse medicine reactions [63]. In a similar development, Appiah et al [55] supported the development of caller tunes to encourage blood donation, while they [56] opined that those who thought mobile caller tunes could improve medicine adherence were higher among those who approved the application. Universally, the use of mobile phones among health practitioners in refining health care has gained more attention [64]. Andreatta et al [49] suggested that birth attendants may be trained to use mobile phones to report and transfer postpartum hemorrhage data.

Furthermore, the findings of Pop-Eleches et al [44] suggested that reminders in the form of SMS text messages on mobile phones could improve response to antiretroviral therapy in regions with scarce resources. Similarly, Raifman et al [43] opined that receiving mobile SMS text messages can advance the use of antimalarial treatment. The study of Babatunde et al [34] also corroborated that, potentially, mHealth may improve access to health care services. In another study, L'Engle et al [45] engaged mobile phones in improving HIV care and treatment. However, the study was suspended due to issues with the financing source. Similarly, Ishola and Chipps [33] found out that, mHealth could confer psychological flexibility if introduced into the prevention of mother-child HIV transmission. These findings agree with those of Dzansi et al [65], who concluded that mobile phones improved health outcomes in low- and middle-income settings.

Conversely, Stephani et al [48] posited that necessary resources and training are required for the effective adoption of mHealth solutions. He found out that patients at the diabetic center had a good mentality about mobile phones but were unfamiliar with them. The conclusion of Kenny et al [36] also indicates that despite the role of mobile phones in improving the health of sick children, health care providers have identified challenges limiting its widespread adoption. The findings of Gurupur and Wan [66] also concur with these findings.

Mobile technology has not only been effective in general health but also sexual and reproductive health. Based on findings from Rokicki et al [47], text-messaging initiatives have the potential to meaningfully improve reproductive health awareness and abate pregnancy. Similarly, Laing et al [54] reported that communications via mobile phones have made a positive impact on monitoring birth outcomes, and Otu et al [37] also opined that mHealth can transform the delivery of sexual reproductive health.

### Theme 2: Tele- and e-Based Interventions

In rural areas, the cost of visiting hospitals often can be high. The COVID-19 pandemic made physical contact dangerous, and lockdowns became inevitable [67]. This has made the preference for telemedicine increase. Based on the outcome of Webb et al [9], he opined that tele-audiology could enhance better access to professional hearing health treatments. In a similar line, the use of telemedicine for equal access to health was evaluated by Bagayoko et al [59]. In the same vein, Bagayoko et al [59] found out that telehealth activities may improve medical diagnostics in cardiology and obstetrics and also the on-site patient monitoring system. It was also reported that health workers who took part in a telepathology program affirmed that the program culminated in increased skills and knowledge about cytopathology [40].

A study carried out by Mbemba et al [62] demonstrated the positive impact of telemedicine on the hiring and retention of medical professionals in Mali's rural locations. This aligns with Hicks et al [58], who found that video training supported by eHealth technology is an acceptable technique for improving clinical knowledge and service delivery to primary health workers in Nigeria.

The knowledge and insights of health care professionals (HCPs) who are at the center of health care delivery about telemedicine are key in determining its future [68]. According to the report of Abodunrin and Akande [42], it was submitted that health care professionals have good knowledge of telemedicine and eHealth. Nevertheless, they alluded to limitations in the application of telemedicine and e-Health in Nigeria to cost and dearth of infrastructure. In the same vein, Monsudi et al [38] reported that the importance of telemedicine in West Africa is known among many HCPs. However, only a few of them were aware of its availability at the hospital. Of all the papers reviewed, only Batta and Iwokwagh [60] opined that the use of news and social media serves as a means of transmitting health information. Nonetheless, its benefit has not been fully harnessed in the health care space. This agrees with the findings of Bekalu et al [69].

### Theme 3: Digital Interventions

The report of Tabari [39] maintained the use of low-cost substitutes for printing computerized tomography image scans in developing countries via digital health technology. The outcome of Ebenso et al [53] is that video training and data digitization are promising solutions for promoting changes in maternal-child health care services. Similarly, Akeju et al [57] reported that video training and data digitization improved the level of maternity care delivery and attendance at health facilities. The work of Olu et al [35] reviewed the gains and pains accompanying the implementation of digital health for universal health coverage and proposed a conceptual framework that could make it widely implemented. Interestingly, of all the papers under review, only Ofoegbu et al [46] accessed the impact of education through digital storytelling. According to their study, education through digital storytelling was effective in boosting the risk perception and awareness of HIV among Nigerian adolescents.

## Discussion

### Overview

This review paper examines the impact of digital interventions on medication delivery in West Africa. It highlights the dire state of health care systems in the region and the potential for digital interventions to address these challenges. The study finds that phone-based interventions are the most used, with positive impacts on drug adherence and data reporting. However, there are limitations in study designs, sample sizes, and statistical methods across the reviewed literature. The paper recommends increased training for health care professionals, public awareness campaigns, and government intervention to address infrastructure challenges. Despite its thoroughness, the review acknowledges the uneven distribution of literature across West Africa and calls for further research to provide robust information on digital interventions. Overall, the review provides valuable insights into the current state and future potential of digital interventions in West African health care.

### Major Findings

We carried out a systematic review of the literature that assessed major digital interventions for medication and health care delivery in West Africa. In this study, mobile devices, tele- and electronic tools, and digital devices were used as interventions. The most frequently used is the phone-based intervention. The use of phone-based interventions has gained access into the health care space more than other interventions. Precisely, 30% of the studies under review focused on tele- and e-based tools as a digital health intervention. It is surprising to see that no study reported the use of news, social media, wearable devices, or videoconferencing as a digital intervention for health care delivery. Overall, 17% of the studies under review used digital tools as interventions. Interestingly, video training and data digitization are gradually gaining acceptance in Africa. Also, remarkably, the report of Ofoegbu et al [46] was striking as it focused on addressing a particular challenge among adolescents using digital storytelling.

The second research question, “how well have these interventions been able to impact the health care system among West Africans and what are the challenges limiting its widespread adoption?” was also answered. We discovered that these digital interventions have impacted the health care system among West Africans in the area of drug adherence and reporting mechanisms, leading to improved data sets. This can therefore serve as a teaching aid to both professionals and people, as a public health strategy and in direct care delivery. These studies point out the fact that digital intervention has had a positive impact and will positively impact the future of medication delivery and the health care system in West Africa. However, the paucity of information on the impacts of digital tools as an intervention in the health care space in West Africa calls for concern.

### Critical Appraisal

During the reviewing process, 12 qualitative papers were included and 17 quantitative papers were reviewed. We carried out a critical evaluation with a focus on the papers' strengths and flaws. The evaluation followed the themes that were used in the systematic review. The first theme emphasized phone-based intervention. After reading the work of Adedeji et al [50], it became clear that the novelty of the study was that it was not self-reported. Instead, the authors tracked the purchase of antimalaria medications from 4 patent and proprietary medicine stores and 4 community pharmacies in a particular Nigerian community. The study, however, cannot be extrapolated to all of Nigeria due to the small sample size, time, and financial efficiency of the study. It is interesting to note that the study by Pop-Eleches et al [44] was one of the first of its kind to use a quantitative approach to demonstrate the positive effects of mHealth in the provision of antiretroviral therapy (HIV/AIDS) care. The power of the analysis may be lowered since the study's scope is too wide and the sample size is small. These restrictions mirrored those of Adedeji et al [50].

Following a critique of Ishola and Chipps [33], the study used a randomized control experiment with empirical data to gauge the effectiveness of a mobile health intervention. Scores from the pre- and post-tests were analyzed. The study's conclusions, however, were constrained in that they could not be generalized; hence, it is important to use caution when applying research findings. The test results are constrained by the inadequacies of the measures used. Another study by Kenny et al [36] looked at the difficulties in adopting mHealth from the viewpoint of primary health care workers. Nevertheless, because a specific case study project was chosen, the generalization may be difficult. Furthermore, the hypothesis was not empirically validated, which further restricted the findings' ability to be generalized. In a similar line, the findings of Andreatta et al [49] were deemed strong in that they looked into the significance of cell phones for reporting postpartum hemorrhage data by professional and traditional delivery attendants in rural Africa. The survey data, however, was not quantified using the proper statistical methods for the post-test design. Reporting solely on how the attendant adhered to the established cell phone usage protocol is unreliable. This implies that a significant portion of the findings on digital health interventions in Africa would necessitate a suitable experimental design and precise statistical methods right from the start.

On the other hand, after delving deeply into the conclusions of Kukula et al [52], we realized that the study's strength was a very large sample size and a powerful quantitative analysis that was used to analyze the data gathered. The project could not, however, be completed without financial support from an external institution due to its cost implications. Similar to this, the study by Appiah et al [55] received excellent marks because it tested the hypothesis using structural equation modeling, a powerful statistical technique. However, because the study was carried out in a specific hospital in Ghana, generalizing about rural and urban settings, much alone Sub-Saharan Africa, is inappropriate. Similarly, Rokicki et al [47] randomly assigned their subjects (adolescent girls) into groups using a high sample size and a computer-generated random number. Despite this, all information in the survey was self-reported; participants in trials might have been more tempted to hide their sexual behavior. The survey does not include adolescent girls in the rural sector and is primarily focused on Ghana’s urban areas. Additionally, starting with their initial antenatal appointments, Laing et al [54] followed pregnant women and kept track of any potentially harmful birth outcomes. In order to track stillbirths and neonatal fatalities, the author used a mobile telephone follow-up regardless of whether the expectant woman gave delivery inside or outside of a hospital. However, the outcome of an observational study is somewhat unreliable compared with resolving empirical evidence.

We evaluated other studies and discovered that these had limitations in their outcomes, making them differ from studies with limitations due to study designs, sample sizes, and other statistical methods. Another study [45] was notable for being the first to use a clinical trial to investigate the effects of mobile health on HIV prevention and care among PLHIV in a large-scale public sector context in Ghana. It was self-reported adherence to antiretroviral therapy, though, and this has notable limitations. This might take the place of laboratory tests or objective measurements from doctors, pharmacists, or lab scientists. Additionally, Raifman et al [43] should be commended for seeking the participants' consent prior to conducting research. This was accomplished using flyers to find volunteers for an intervention and a self-enrolled mobile health service. As a result, those unwilling to engage in the study were not included. Even though they were willing to join, individuals without mobile phones were sadly unable to receive SMS text messages. Similar to this, the findings of Babatunde et al [34] emphasized how rural areas have limited access to mHealth, which hinders their ability to achieve Universal Health Coverage due to issues like inadequate electricity, bad data, limited internet connection, expensive mobile phones, etc. However, the research might be used as more proof rather than a description of the issue and possible solutions, such as mHealth. In a similar line, the findings of Appiah et al from 2021 were convincing about the development of the ATM to enhance medication adherence in Ghana. However, the study does not take into account that moderators can affect the findings. The study setting (diabetes clinic, Kumasi, Ghana) has significant promise for mHealth, and the methods used to analyze the survey results were supported by science, according to Stephani et al [48]. However, only patients who were present in the clinic at the time of the survey were interviewed, and because the responses were self-reported, the internal validity and reliability of the responses could not be determined because the sample selection technique could not accommodate patients outside the clinic. The study by Appiah et al [51] had a strong point in that it examined the significance of mHealth technology for the promotion of reporting ADRs; however, factors that could impact the production and usage of caller tunes to telecommunications were not considered. The findings of Otu et al [37] addressed a specific aspect of health care delivery and were considered and applied to a Nigerian context; however, not much evidence was provided on why, what form, or how the mHealth adoption process can be implemented.

Furthermore, we reviewed holistically the articles that were discussed under theme 2, which focused on tele- and e-based intervention. The results of the study by Mbemba et al [62] were deemed to be strong in that it evaluated the perceived impact of telemedicine on the hiring and retention of medical professionals in rural locations in Mali; however, the study did not include the actual demonstration of telehealth in recruiting and retaining professional workers in a remote area; rather, it only sought the perception of the health care workers in a remote area. In a similar vein, an assessment by Bagayoko et al [59] found that the strength of the study was in the fact that it examined the economic benefit of telehealth in 3 different district hospitals in Mali. To increase the accuracy of the outcome, however, there was hypothesis testing. Frequency and percentage alone are insufficient. In a similar vein, the study by Monsudi et al [38] deserves praise for examining the availability and awareness of telemedicine from the specific employees in a particular region of Nigeria. Although it was hampered by its departure from its intended topic by discussing the number of attendees rather than concentrating solely on addressing the causes for people's ignorance of telemedicine's presence in the hospital. Therefore, the study cannot be extrapolated to the entirety of Nigeria, which conflicts with findings from studies conducted in a different part of the nation. The study conducted by Malami [40] portrayed that the evaluation of the effects of telepathology training on ongoing cytology education was another strength. The study did not, however, carry out the cytology training itself. It only examined the success of earlier telepathology and recommended that cytology training follow suit.

The study by Batta and Iwokwagh [60] was interesting since it showed that the advantages of news and social media have not been sufficiently investigated. The majority of teaching hospitals and specialty clinics use it for publicity. Its shortcomings were that it advocated for Nigeria to adopt the concept of news and social media platforms without outlining the precise steps that may be taken to maximize their usefulness. Similarly, the investigation of the viability and acceptance of eHealth technologies in enhancing service delivery and performance of primary health workers in Nigeria was a strength of the study conducted by Hicks et al [58]. But just 3 Nigerian states were examined in the study. This can be a result of a financial problem. Additionally, there was no mention of reaching the states' rural areas. In contrast, the study by Abodunrin and Akande [42] has the advantage of identifying the potential causes—such as a lack of reliable electricity or internet access or unstable political conditions—that might be impeding Nigeria's adoption of e-Health and telemedicine to the fullest. However, the study was conducted in a specific state in Nigeria. Hence, generalizing is a problem here. The assessment of Bagayoko et al [61] revealed a summary of its strength, which was specific to using Réseau en Afrique Francophone pour la Télémédecine network to improve health care delivery in Mali. The shallowness of the findings and conclusion on the use of Réseau en Afrique Francophone pour la Télémédecine limited the study.

Next, we assessed the pieces that fell within theme 3. The power of the work by Olu et al [35] was demonstrated by the way it presented a range of benefits and drawbacks of Africans adopting digital technology in order to develop a conceptual framework for implementation. The research, however, is very theoretical and general. Using Nigerian college students as a case study, the essay expertly examined the impact of digital storytelling on HIV perception and knowledge [46]. Nevertheless, the sample size is modest when you evaluate Nigeria's entire state from a broad perspective. The study's focus is also restricted to just one particular area of Nigeria. Thus, in a similar discussion of constraints relating to design and analytical techniques, ref. [46] was also included.

On the other hand, the constraints of a few more studies affected the results. For instance, Tabari [39] proposed a low-cost alternative way of producing computerized tomography scan images in the absence of a dedicated camera. This is a really solid argument for the piece. The analysis of the created low-cost computerized topographical image, however, is not sufficiently detailed for replication. As a result, the study is only descriptive and constrained. In a similar vein, Ebenso et al [53] conducted a study in rural Nigeria (3 distinct states) to improve the provision of maternal and child health care. However, the study's focus is limited to rural areas with few resources. Urban areas might not be aware that digital health interventions are being used. In conclusion, the findings by Akeju et al [57] made a solid case for the distribution of video training and data digitization interventions to 62 health care facilities over a 2-year period (2017-2019) in areas lacking SatCom and 3G mobile networks. Although the report referenced southwest Nigeria, it only included information on one of the 5 Southwestern states' rural health facilities. Therefore, the study does not explore what the government may do to enhance eHealth applications in rural areas. These strengths and flaws mainly focus on experimental designs, statistical tools used, study size, and outcomes. Hence, it is pertinent to consider using the right sample size and statistical tools for a better outcome [70,71].

### Overall Integrated Findings and Comparison With Previous Research

Following the critical appraisal, there is also guidance on the overall integrated findings of this review, which informs recommendations that guide evidence-based policy and agenda for future research. According to Stern et al [72] and Lizarondo et al [73], where the core review question uses a mix of qualitative and quantitative papers, authors can use the convergent integrated approach, which allows for transformation of data and for capturing integrated findings from the review. Consequently, our review showed that 53.3% of the studies under review used the phone as an intervention. A previous study [74] conducted among community pharmacists showed that 69.2% of the studies reviewed used the telephone as a digital intervention, while another study [75] showed that 78.9% of the clinical pharmacists preferred the use of the telephone to other digital intervention tools. Both studies agree with the result of our review, as they pitched the telephone as the most regularly used digital health intervention.

### Strengths and Limitations

A major strength of this review is the huge and productive efforts put into verifying reasonable parameters. There are also areas of significant input as well as areas of improvement for papers reviewed while we surveyed various digital health interventions. Additionally, the research approach used is another strong point of this review. This opened the researchers to diverse instruments, models, and parameters used to evaluate digital interventions for medication and health care delivery in West Africa.

However, various limitations are worthy of note. First and foremost, this literature review only focuses on a few West African countries. This means that there are many countries that were not included while conducting this review. The focus on a few West African countries limits the generalizability of findings, highlighting the need for broader regional studies. Furthermore, the authors were also constrained by the word count limit, thus, the themes and subthemes could not be adequately explored, while the various columns in the critical review table showing methodology and areas of strengths and weaknesses can be improved. Furthermore, there are very limited studies in West Africa on this subject at the moment. A meta-analysis would be a consideration for future studies, but with more focus on Sub-Saharan Africa to do a deeper and in-depth search on a larger sample/region size. Although a thorough check of each paper included in the review has been conducted following existing quality checks such as CASP checklists, other formal checks such as Joanna Briggs Institute’s critical appraisal tools can also be beneficial.

### Conclusions

This review paper is the first systematic review of the impacts of digital interventions on medication delivery in West Africa. To ensure we do not miss any relevant articles, we followed the recommendations of the PRISMA statement. This review shows that in West Africa, the availability of literature across West Africa is not even. Thus, more research needs to be done in all regions of West Africa to provide robust information on the digital interventions for medication delivery in West Africa. Overall, we found that phone-based interventions and tele- and e-based interventions were largely more helpful, which is a strong basis to recommend the sustained use of these interventions. Moreover, only a little evidence is available about the solutions to the challenges limiting the use of digital interventions. The critical appraisal of existing studies underscores the need for robust research methodologies and rigorous statistical analysis to ensure the reliability and generalizability of findings. Many studies reviewed exhibited limitations in sample size, study design, and analytical techniques, highlighting the importance of addressing these shortcomings in future research endeavors. Thus, there is a need for comprehensive research to first identify innovative interventions that will be acceptable both in rural and urban areas, proffer solutions to the menace of poor implementation of these interventions, and formulate policies that would encourage a widespread integration of these interventions into the health care system in West Africa.

Policy interventions are crucial to creating an enabling environment for the widespread adoption of digital interventions. Governments and relevant stakeholders should prioritize investments in health care infrastructure and technology while also formulating policies that incentivize the use of digital solutions. Moreover, collaboration between governments, health care institutions, and technology providers is essential to ensure the successful implementation and sustainability of digital health care initiatives in West Africa.

### Recommendations

The main recommendation of this study is that the use of digital interventions for medication delivery needs to be significantly improved. Following the papers reviewed, it has been shown clearly that individuals, patients, and health care professionals have adopted some interventions, while some have not been implemented at all. There is, therefore, a need for a holistic implementation of digital intervention tools in the health care space in West Africa.

First, health care professionals need to be trained on the technicalities necessary to integrate digital interventions into health care delivery in West Africa. This is in line with the findings of Feroz et al [76]. Furthermore, people should be sensitized about the use of DI in health care delivery both in rural and urban areas. In addition, Abodunrin and Akande [42] pinpointed the possible reasons for the poor adoption of digital interventions, such as poor power supply, internet service, the poor state of infrastructure, and so on. Therefore, this review again recommends that the government and other key agencies involved in proffering solutions to these should not hesitate to. Finally, platforms must be established for training HCPs on managing the technicalities involved, sensitizing the users, and formulating policies that would encourage a widespread integration of these interventions into the health care system in West Africa.

## Appendix

Multimedia Appendix 1 Description of included studies.

Multimedia Appendix 2 PRISMA (Preferred Reporting Items for Systematic Reviews and Meta-Analyses) 2020 checklist.

Multimedia Appendix 3 Number of papers per intervention. Source: Authors’ compilation.

## Abbreviations

CASP: Critical Appraisal Skills Programme
HCP: health care professional
mHealth: mobile health
PRISMA: Preferred Reporting Items for Systematic Reviews and Meta-Analyses
SatCom: satellite communications

## Data Availability

All data generated or analyzed during this study are included in this published article and its supplementary information files. They are made publicly available.

## References

[1] Valverde S (2016). Major transitions in information technology. Philos Trans R Soc Lond B Biol Sci.

[2] Michie S, Yardley L, West R, Patrick K, Greaves F (2017). Developing and evaluating digital interventions to promote behavior change in health and health care: recommendations resulting from an international workshop. J Med Internet Res.

[3] Awad A, Trenfield SJ, Pollard TD, Ong JJ, Elbadawi M, McCoubrey LE, Goyanes A, Gaisford S, Basit AW (2021). Connected healthcare: improving patient care using digital health technologies. Adv Drug Deliv Rev.

[4] Lee P, Aikens J, Richardson C, Singer D, Kullgren J, Kirch M, Solway E, Smith E, Malani P (2020). Mobile health app use among older adults. University of Michigan National Poll on Healthy Aging.

[5] Ventola CL (2014). Mobile devices and apps for health care professionals: uses and benefits. Pharm Therap.

[6] Koivunen M, Niemi A, Hupli M (2015). The use of electronic devices for communication with colleagues and other healthcare professionals - nursing professionals' perspectives. J Adv Nurs.

[7] Pal K, Dack C, Ross J, Michie S, May C, Stevenson F, Farmer A, Yardley L, Barnard M, Murray E (2018). Digital health interventions for adults with type 2 diabetes: qualitative study of patient perspectives on diabetes self-management education and support. J Med Internet Res.

[8] Kassavou A, Houghton V, Edwards S, Brimicombe J, Sutton S (2019). Development and piloting of a highly tailored digital intervention to support adherence to antihypertensive medications as an adjunct to primary care consultations. BMJ Open.

[9] Webb J, Peerbux S, Smittenaar P, Siddiqui S, Sherwani Y, Ahmed M, MacRae H, Puri H, Bhalla S, Majeed A (2020). Preliminary outcomes of a digital therapeutic intervention for smoking cessation in adult smokers: randomized controlled trial. JMIR Ment Health.

[10] Raijada D, Wac K, Greisen E, Rantanen J, Genina N (2021). Integration of personalized drug delivery systems into digital health. Adv Drug Deliv Rev.

[11] Hare AJ, Chokshi N, Adusumalli S (2021). Novel digital technologies for blood pressure monitoring and hypertension management. Curr Cardiovasc Risk Rep.

[12] Odone A, Gianfredi V, Sorbello S, Capraro M, Frascella B, Vigezzi GP, Signorelli C (2021). The use of digital technologies to support vaccination programmes in europe: state of the art and best practices from experts' interviews. Vaccines (Basel).

[13] Marthick M, McGregor D, Alison J, Cheema B, Dhillon H, Shaw T (2021). Supportive care interventions for people with cancer assisted by digital technology: systematic review. J Med Internet Res.

[14] Bevilacqua R, Casaccia S, Cortellessa G, Astell A, Lattanzio F, Corsonello A, D'Ascoli P, Paolini S, Di Rosa M, Rossi L, Maranesi E (2020). Coaching through technology: a systematic review into efficacy and effectiveness for the ageing population. Int J Environ Res Public Health.

[15] Ibeneme S, Ukor N, Ongom M, Dasa T, Muneene D, Okeibunor J (2020). Strengthening capacities among digital health leaders for the development and implementation of national digital health programs in nigeria. BMC Proc.

[16] Giravi HY, Biskupiak Z, Tyler LS, Bulaj G (2022). Adjunct digital interventions improve opioid-based pain management: impact of virtual reality and mobile applications on patient-centered pharmacy care. Front Digit Health.

[17] Ibrahim MS, Mohamed Yusoff H, Abu Bakar YI, Thwe Aung MM, Abas MI, Ramli RA (2022). Digital health for quality healthcare: a systematic mapping of review studies. Digit Health.

[18] Oleribe OO, Momoh J, Uzochukwu BS, Mbofana F, Adebiyi A, Barbera T, Williams R, Taylor-Robinson SD (2019). Identifying key challenges facing healthcare systems in Africa and potential solutions. Int J Gen Med.

[19] WHO (2013). African Region Health Report.

[20] (2022). Health care in Africa: IFC report sees demand for investment. International Finance Corporation.

[21] Wale-Oshinowo BA, Omobowale AO, Adeyeye MM, Lebura S (2022). Least developed countries in Africa. The Palgrave Encyclopedia of Global Security Studies.

[22] Page MJ, McKenzie JE, Bossuyt PM, Boutron I, Hoffmann TC, Mulrow CD, Shamseer L, Tetzlaff JM, Akl EA, Brennan SE, Chou R, Glanville J, Grimshaw JM, Hróbjartsson A, Lalu MM, Li T, Loder EW, Mayo-Wilson E, McDonald S, McGuinness LA, Stewart LA, Thomas J, Tricco AC, Welch VA, Whiting P, Moher D (2021). The PRISMA 2020 statement: an updated guideline for reporting systematic reviews. Br Med J.

[23] Wienert J, Jahnel T, Maaß L (2022). What are digital public health interventions? First steps toward a definition and an intervention classification framework. J Med Internet Res.

[24] Nievas SB, García DS, Fernández AA, Bonillo PA, Parrón CT (2019). eHealth: advantages, disadvantages and guiding principles for the future. J Med Internet Res.

[25] Oh H, Rizo C, Enkin M, Jadad A (2005). What is eHealth (3): a systematic review of published definitions. J Med Internet Res.

[26] Shaw T, McGregor D, Brunner M, Keep M, Janssen A, Barnet S (2017). What is eHealth (6)? Development of a conceptual model for eHealth: qualitative study with key informants. J Med Internet Res.

[27] Boogerd EA, Arts T, Engelen LJ, van de Belt TH (2015). "What Is eHealth": time for an update?. JMIR Res Protoc.

[28] Iacono T, Stagg K, Pearce N, Hulme Chambers A (2016). A scoping review of Australian allied health research in ehealth. BMC Health Serv Res.

[29] Pagliari C, Sloan D, Gregor P, Sullivan F, Detmer D, Kahan JP, Oortwijn W, MacGillivray S (2005). What is eHealth (4): a scoping exercise to map the field. J Med Internet Res.

[30] Davis TL, DiClemente R, Prietula M (2016). Taking mHealth forward: examining the core characteristics. JMIR Mhealth Uhealth.

[31] Fatehi F, Samadbeik M, Kazemi A (2020). What is digital health? Review of definitions. Stud Health Technol Inform.

[32] Zeeb H, Pigeot I, Schüz B, Leibniz-Wissenschafts Campus Digital Public Health Bremen (2020). Digital public health-an overview. Bundesgesundheitsblatt Gesundheitsforschung Gesundheitsschutz.

[33] Ishola AG, Chipps J (2015). The use of mobile phones to deliver acceptance and commitment therapy in the prevention of mother-child HIV transmission in Nigeria. J Telemed Telecare.

[34] Babatunde AO, Abdulkareem AA, Akinwande FO, Adebayo AO, Omenogor ET, Adebisi YA, Ilesanmi EB (2021). Leveraging mobile health technology towards achieving universal health coverage in Nigeria. Public Health Pract (Oxf).

[35] Olu O, Muneene D, Bataringaya JE, Nahimana MR, Ba H, Turgeon Y, Karamagi HC, Dovlo D (2019). How can digital health technologies contribute to sustainable attainment of universal health coverage in Africa? A perspective. Front Public Health.

[36] Kenny G, O’Connor Y, Eze E, Ndibuagu E, Heavin C (2017). A ground-up approach to mHealth in Nigeria: a study of primary healthcare workers’ attitude to mHealth adoption. Proc Comput Sci.

[37] Otu A, Ukpeh I, Okuzu O, Yaya S (2021). Leveraging mobile health applications to improve sexual and reproductive health services in Nigeria: implications for practice and policy. Reprod Health.

[38] Monsudi KF, Ayanniyi AA, Oguntunde OO (2012). Awareness and practice of telemedicine among staff of the federal medical centre at Birnin Kebbi, Nigeria. J Telemed Telecare.

[39] Tabari AM (2007). Low-cost printing of computerised tomography (CT) images where there is no dedicated CT camera. J Telemed Telecare.

[40] Malami SA (2008). Use of telemedicine for cytology training in Africa. J Telemed Telecare.

[41] Swanepoel DW, Olusanya BO, Mars M (2010). Hearing health-care delivery in sub-Saharan Africa--a role for tele-audiology. J Telemed Telecare.

[42] Abodunrin OL, Akande TM (2010). Knowledge and perception of e-health and telemedicine among health professionals in lautech teaching hospital, Osogbo, Nigeria. Int J Health Res.

[43] Raifman JRG, Lanthorn HE, Rokicki S, Fink G (2014). The impact of text message reminders on adherence to antimalarial treatment in northern Ghana: a randomized trial. PLoS One.

[44] Pop-Eleches C, Thirumurthy H, Habyarimana JP, Zivin JG, Goldstein MP, de Walque D, MacKeen L, Haberer J, Kimaiyo S, Sidle J, Ngare D, Bangsberg DR (2011). Mobile phone technologies improve adherence to antiretroviral treatment in a resource-limited setting: a randomized controlled trial of text message reminders. AIDS.

[45] L'Engle KL, Green K, Succop SM, Laar A, Wambugu S (2015). Scaled-up mobile phone intervention for HIV care and treatment: protocol for a facility randomized controlled trial. JMIR Res Protoc.

[46] Ofoegbu TO, Otu MS, Christopher I, Uche A, Nwabuko LO, Ebere I, Dike IC, Ngozi O, Chinedozie U, Muhammed A (2020). Impact of an educational digital storytelling intervention on HIV risk perception among Nigerian adolescents. J Int Med Res.

[47] Rokicki S, Cohen J, Salomon JA, Fink G (2017). Impact of a text-messaging program on adolescent reproductive health: a cluster-randomized trial in Ghana. Am J Public Health.

[48] Stephani V, Opoku D, Otupiri E (2020). Determining the potential of mobilephone-based health interventions in kumasi, Ghana. Ghana Med J.

[49] Andreatta P, Debpuur D, Danquah A, Perosky J (2011). Using cell phones to collect postpartum hemorrhage outcome data in rural Ghana. Int J Gynaecol Obstet.

[50] Adedeji AA, Sanusi B, Tella A, Akinsanya M, Ojo O, Akinwunmi MO, Tikare OA, Ogunwande IA, Ogundahunsi OA, Ayilara OO, Ademola TT, Fehintola FA, Ogundahunsi OAT (2011). Exposure to anti-malarial drugs and monitoring of adverse drug reactions using toll-free mobile phone calls in private retail sector in Sagamu, Nigeria: implications for pharmacovigilance. Malar J.

[51] Appiah B, Poudyal A, Burdine JN, Asamoah-Akuoko L, Anum DA, Kretchy IA, Sabblah G, Dodoo ANO, McKyer ELJ (2019). Factors that influence the intention to use mobile phone caller tunes for patient reporting of adverse drug reactions: a qualitative study. Ther Adv Drug Saf.

[52] Kukula VA, Dodoo AAN, Akpakli J, Narh-Bana SA, Clerk C, Adjei A, Awini E, Manye S, Nagai RA, Odonkor G, Nikoi C, Adjuik M, Akweongo P, Baiden R, Ogutu B, Binka F, Gyapong M (2015). Feasibility and cost of using mobile phones for capturing drug safety information in peri-urban settlement in Ghana: a prospective cohort study of patients with uncomplicated malaria. Malar J.

[53] Ebenso B, Okusanya B, Okunade K, Akeju D, Ajepe A, Akaba GO, Yalma RM, Dirisu O, Tukur J, Abdullahi MK, Okuzu O, Allsop MJ (2021). What are the contextual enablers and impacts of using digital technology to extend maternal and child health services to rural areas? Findings of a qualitative study from Nigeria. Front Glob Womens Health.

[54] Laing S, Remmelzwaal K, Cooper M, N'Dow J (2020). Mobile telephone follow-up to ascertain birth outcomes in The Gambia. Telemed J E Health.

[55] Appiah B, Burdine JN, Aftab A, Asamoah-Akuoko L, Anum DA, Kretchy IA, Samman EW, Appiah PB, Bates I (2018). Determinants of intention to use mobile phone caller tunes to promote voluntary blood donation: cross-sectional study. JMIR Mhealth Uhealth.

[56] Appiah B, Kretchy IA, Yoshikawa A, Asamoah-Akuoko L, France CR (2021). Perceptions of a mobile phone-based approach to promote medication adherence: a cross-sectional application of the technology acceptance model. Explor Res Clin Soc Pharm.

[57] Akeju D, Okusanya B, Okunade K, Ajepe A, Allsop MJ, Ebenso B (2022). Sustainability of the effects and impacts of using digital technology to extend maternal health services to rural and hard-to-reach populations: experience from Southwest Nigeria. Front Glob Womens Health.

[58] Hicks JP, Allsop MJ, Akaba GO, Yalma RM, Dirisu O, Okusanya B, Tukur J, Okunade K, Akeju D, Ajepe A, Okuzu O, Mirzoev T, Ebenso B (2021). Acceptability and potential effectiveness of eHealth tools for training primary health workers from Nigeria at scale: mixed methods, uncontrolled before-and-after study. JMIR Mhealth Uhealth.

[59] Bagayoko CO, Traoré D, Thevoz L, Diabaté S, Pecoul D, Niang M, Bediang G, Traoré ST, Anne A, Geissbuhler A (2014). Medical and economic benefits of telehealth in low- and middle-income countries: results of a study in four district hospitals in mali. BMC Health Serv Res.

[60] Batta HE, Iwokwagh NS (2015). Optimising the digital age health-wise: utilisation of new/social media by Nigerian teaching hospitals. Proc Soc Behav Sci.

[61] Bagayoko CO, Anne A, Fieschi M, Geissbuhler A (2011). Can ICTs contribute to the efficiency and provide equitable access to the health care system in sub-Saharan Africa? The Mali experience. Yearb Med Inform.

[62] Mbemba GIC, Bagayoko CO, Gagnon MP, Hamelin-Brabant L, Simonyan DA (2016). The influence of a telehealth project on healthcare professional recruitment and retention in remote areas in Mali: a longitudinal study. SAGE Open Med.

[63] NPS Medicinewise. National Prescribing Service, Medicine wise.

[64] Kalinga A, Munga M, Ngenya A, John W, Kisoka W, Oriyo N, Mutalemwa P, Mandara W, Masagati L, Ogondiek J, Korir P, Klarmann-Schulz U, Horn S, Kroidl I, Debrah A, Hoerauf A, Mwingira U (2022). The viability of utilising phone-based text messages in data capture and reporting morbidities due to lymphatic filariasis by community health workers: a qualitative study in Kilwa district, Tanzania. BMC Health Serv Res.

[65] Dzansi G, Chipps J, Lartey M (2022). Use of mobile phone among patients with HIV/AIDS in a low-middle income setting: a descriptive exploratory study. Behav Inf Technol.

[66] Gurupur VP, Wan TTH (2017). Challenges in implementing mHealth interventions: a technical perspective. mHealth.

[67] Haleem A, Javaid M, Singh RP, Suman R (2021). Telemedicine for healthcare: capabilities, features, barriers, and applications. Sens Int.

[68] Ayatollahi H, Sarabi FZP, Langarizadeh M (2015). Clinicians' knowledge and perception of telemedicine technology. Perspect Health Inf Manag.

[69] Bekalu MA, McCloud RF, Viswanath K (2019). Association of social media use with social well-being, positive mental health, and self-rated health: disentangling routine use from emotional connection to use. Health Educ Behav.

[70] Ali Z, Bhaskar SB (2016). Basic statistical tools in research and data analysis. Indian J Anaesth.

[71] Andrade C (2020). Sample size and its importance in research. Indian J Psychol Med.

[72] Stern C, Lizarondo L, Carrier J, Godfrey C, Rieger K, Salmond S, Apóstolo J, Kirkpatrick P, Loveday H (2020). Methodological guidance for the conduct of mixed methods systematic reviews. JBI Evid Synth.

[73] Lizarondo L, Stern C, Apostolo J, Carrier J, de Borges K, Godfrey C, Kirkpatrick P, Pollock D, Rieger K, Salmond S, Vandyk A, Loveday H (2022). Five common pitfalls in mixed methods systematic reviews: lessons learned. J Clin Epidemiol.

[74] Crilly P, Kayyali R (2020). A systematic review of randomized controlled trials of telehealth and digital technology use by community pharmacists to improve public health. Pharmacy (Basel).

[75] Park T, Muzumdar J, Kim H (2022). Digital health interventions by clinical pharmacists: a systematic review. Int J Environ Res Public Health.

[76] Feroz AS, Khoja A, Saleem S (2021). Equipping community health workers with digital tools for pandemic response in LMICs. Arch Public Health.

