# Supplementary material for: Digital Interventions for Managing Medication and Health Care Service Delivery in West Africa: Systematic Review
Source: J Med Internet Res. 2024 Oct 9;26:e44294. doi: 10.2196/44294 (PMC11499747; doi:10.2196/44294)
Supplement: Multimedia Appendix 1 [file jmir_v26i1e44294_app1.docx]

### **Multimedia Appendix 1. Description of included studies**

| S/N | Author | Journal | Type of Health Issue and Health Technology | Research Approach/Method | Main Findings | Critical Appraisal |
| --- | --- | --- | --- | --- | --- | --- |
| 1 | [33] | Journal of Telemedicine and Telecare | - Mother-child HIV transmission  - mHealth (weekly mobile phone messages) | - Solomon four-group (two intervention and two control groups) randomised design  - Action and Acceptance Questionnaire (AAQ-II) scores | There is a potential for psychological flexibility if mHealth is introduced, accepted, and committed to managing therapy programmes. | **Strengths:**  -The study uses an empirically data-driven randomised control experiment to test the impact of a mHealth intervention  - Pre- and post-tests scores were conducted.  **Limitations:**  -Results cannot be generalised and the implication of research findings should be done with caution  - Measures used also have weaknesses which limit the tests scores |
| 2 | [34] | Public Health in Practice | - Universal Health Coverage  - Mobile health technology | Exploratory Research | mHealth has the potential to enhance access to healthcare services | **Strengths:**  - Highlights limitation of rural areas to mHealth which affects the possibility of achieving UHC such as poor power supply, poor data, limit to internet access, high cost of mobile phones, etc  **Limitations:**  - The research can be further evidenced rather than descriptive of the problem and potential solutions i.e. mHealth |
| 3 | [35] | Frontiers in Public Health | - Universal Health Coverage  - Digital Health | Exploratory Research | There is an unclear conceptual framework that can help model digital health interventions in Africa | **Strengths:**  - Presents a mix of challenges and benefits of digital adoption in Africa to create a conceptual framework for implementation  **Limitations:**  - The research is theoretical and could have presented a case study of digital health device  - Collapses Africa’s ‘ambitus universal health coverage as its focus. However, this is too broad. |
| 4 | [36] | Procedia Computer Science | - Sick children  - Mobile Health (Mobile Phones for Assessing, Classifying and Treating) | - Qualitative exploratory study  - Ground-Up Approach | The users of mHealth for enhancing health outcomes advocates for communicatee-wide education to reduce negative views of mHealth | **Strengths:**  - The study examines the challenges of mHealth adoption from a primary Healthcare Workers’ perspective.  **Limitations:**  - Generalisation may be challenging as a specific case study project was used.  - The hypothesis is not tested empirically which also limits the generalisation of the findings. |
| 5 | [37] | Reproductive Health | - Sexual and reproductive health services  - Mobile health applications | - Exploratory Research | mHealth has the potential to significantly revolutionise the delivery of sexual reproductive health in Nigeria if policy changes in technological adaptation can be made. | **Strengths:**  - A specific aspect of healthcare delivery is considered and applied to a Nigerian context.  **Limitations:**  - Not much evidence or clarity on why what form or how the mHealth adoption process can be implemented |
| 6 | [38] | Journal of Telemedicine and Telecare | - General Healthcare  - Telemedicine | -Structured Questionnaires  -Exploratory Research | - While the essence of telemedicine was known to most of the staff, very few of them were aware of its availability at the hospital.  - As a result, there is a need to increase healthcare personnel's understanding of telemedicine and to give them training and information about its advantages | **Strengths:**  - The study examined the awareness and availability of telemedicine from the specific staff in a specific region in Nigeria.  **Limitations:**  - The study deviates from its aim by talking and referencing the number of attendees rather than focusing entirely on discussing the reasons for West Africa unawareness of telemedicine’s availability in the hospital.  - The study cannot be generalized to the whole of Nigeria, and hence contradicting other studies in another region of the country. |
| 7 | [39] | Journal of Telemedicine and Telecare | - CT image scan  - Digital health | - Descriptive | - The use of low-cost CT image scans applies to other imaging modalities, such as ultrasound and MRI.  - It can be emulated elsewhere in developing countries (like Nigeria) where resources and technical skills are scarce. | **Strengths:**  - In the absence of a dedicated camera, the study developed a low-cost alternative method of printing computerised tomography (CT) scan images.  **Limitations:**  -The discussion of the developed low-cost computerized topography image is not detailed enough for replication.  - The study is only descriptive and limited |
| 8 | [40] | Journal of Telemedicine and Telecare | - Cytology training  - Telemedicine (slide projectors, multiple telephone lines and amplifier systems) | - Structured questionnaire  - Qualitative approach | The participants reported that the telepathology programme increased their skills and knowledge in cytopathology | **Strengths:**  -The study surveyed to evaluate the telepathology impact training on continuing education in cytology.  **Limitations:**  - The study does not conduct the cytology training itself. It only reviewed the effectiveness of the previous telepathology and advised the same for cytology training as well. |
| 9 | [42] | International Journal of Health Research | - General Healthcare  -Telemedicine, e-Health | - Cross-sectional survey  -Semi-structured questionnaire | - Healthcare professionals have good knowledge of telemedicine and e-Health in delivering healthcare services.  - However, believed that financial constraints and poor state infrastructures are limiting the application of telemedicine and e-Health in Nigeria. | **Strengths:**  - This study pinpointed the possible reasons, such as poor power supply, internet service, and political instability, hindering the full application of e-Health and telemedicine in Nigeria.  **Limitations:**  - The study was conducted in a specific state in Nigeria. Hence, generalizing is a problem here. Moreover, the study does not limit itself to a specific healthcare service. |
| 10 | [43] | PloS one | - Antimalaria treatment  - Mobile text message | - Quantitative technique  - Randomized control trial (control and intervention group) | Receiving mobile text messages can improve the treatment of malaria, although it does not alleviate the impact of malaria sickness during the follow-up interview. | **Strengths:**  - The study was carried out with the consent of the participants. This was achieved through a self-enrolled mobile health programme and flyers for recruiting the participants for an intervention.  - Hence, people not willing to participate in the study were excluded from the study.  **Limitations:**  - Those without mobile phones were not able to receive text messages even if they are willing to participate.  - The use of self-reported adherence as a primary outcome measure also limits the study. |
| 11 | [44] | AIDS | - Antiretroviral treatment (ART)  -Mobile phone technologies (text message reminder | - Quantitative technique  - Randomized control trial | The study suggested that text messages reminder via mobile phone is a crucial tool for improving the response to ART in resource-limited region, such as Nigeria and other West African countries | **Strengths:**  - The study is among the first of its kind that revealed the beneficial effects of mHealth in ART (HIV/AIDS) care delivery through quantitative technique  **Limitations:**  - The study is too broad. The sample size is low, and this may reduce the power of the analysis. |
| 12 | [45] | JMIR research protocols | - HIV care intervention  - mHealth | - Quantitative study  - Cluster randomized controlled design (intervention vs standard delivery care) | - This cluster-RCT would have generated data on the health consequences, psychological features, and cost-effectiveness of mHealth therapies for PLHIV in public-sector settings.  - However, the trial West Africas suspended because the financing from the President's Emergency Plan for AIDS Relief (PEPFAR), which West Africas initially supporting the research, West Africas questionable.  - This is due to the protocol review and approval process.  - As a result, the research is looking for additional funding sources to continue the intervention program. | **Strengths:**  - The study was the first to use the clinical trial to examine the effects of mHealth for HIV prevention and care among PLHIV in a scaled-up public sector setting in Ghana.  **Limitations:**  -It was a self-reported adherence to antiretroviral treatment which has noteworthy restrictions. This could be substituted for laboratory or objective measures from pharmacists, clinicians, and/or lab scientists. |
| 13 | [46] | The Journal of international medical research | - HIV risk perception  - Educational digital storytelling | - Randomized control trial design  -Quantitative methods | The education digital storytelling West Africas effective in boosting the risk perception and awareness of HIV among Nigerian adolescents. | **Strengths:**  - The study investigated the impact of digital storytelling on HIV perception and knowledge using Nigeria college students as a case study.  **Limitations:**  - The sample size is small considering the broad perspective of the whole state in Nigeria. Moreover, the study’s scope is only limited to one specific region in Nigeria. |
| 14 | [47] | American journal of public health | - General Reproductive Health  - mHealth (Text-messaging programme) | - A Cluster–Randomized Trial. | Text-messaging initiatives have the chance to significantly boost reproductive health awareness and minimize pregnancy risk among sexually active adolescent females. | **Strengths:**  -While using the large sample size, the study used a computer-generated random number to divide adolescent girls from 38 different schools into two interventions group and a control group.  **Limitations:**  -The survey relied entirely on self-reported data. Respondents in trials may have felt more tempted to conceal their sexual conduct. They may have underreported pregnancy as a result of receiving messages encouraging the use of contraceptives to avoid unwanted pregnancy.  -The study is limited to the only urban area in Ghana and does not cover adolescent girls in the rural segment. |
| 15 | [48] | Ghana medical journal | - Non-communicable disease (Diabetes Mellitus)  - mHealth | - Cross-sectional study  - Descriptive design | Patients at the diabetic centre had a good mentality about mobile phones but were unfamiliar with them. Whereas patients experienced a variety of accessibility hurdles to treatment, they anticipate the resources for the efficient and long-term adoption of mHealth solutions such as access to mobile phones and electricity were available. | **Strengths:**  -The environment of the diabetic clinic in Kumasi, Ghana, has great potential for mHealth.  -The analysis procedures of the survey responses were scientifically grounded.  **Limitations:**  -The sample selection technique is a convenience sample, meaning that only patients who were currently in the clinic at the time of the survey were interviewed.  - The responses were self-reported in which case the internal validity and reliability of the responses cannot be ascertained. |
| 16 | [49] | International journal of gynaecology and obstetrics: the official organ of the International Federation of Gynaecology and Obstetrics | - Postpartum haemorrhage  - mHealth (cell phones) | - quantitative design  - test-post-test method | The findings suggest that professional and traditional birth attendants may be trained to utilize mobile phones to transmit health-related outcome measures using a predefined approach – a simple numeric protocol | **Strengths:**  - The study investigates the importance of cell phones by professional and traditional birth attendants in rural Africa for reporting postpartum haemorrhage (PPH) data.  **Limitations:**  -The survey data was not quantified with suitable statistical measures for test-post-test design.  - Reporting just how the attendant followed the predefined protocol of using cell phones is not reliable. |
| 17 | [50] | Malaria journal | - Adverse drug reaction (anti-malaria drugs)  - mHealth (toll-free mobile phone calls) | - Observational study | - The ineffective anti-malaria drugs, particularly monotherapies, are nevertheless freely accessible and often purchased in the study region.  - The cost of continuing to utilize unsuccessful monotherapies may be a consideration.  - The availability of a toll-free telephone line may enhance pharmacovigilance and medication response monitoring in a resource-limited environment. | **Strengths:**  - The study is not self-reported as the authors monitor the purchase of anti-malaria drugs from Community Pharmacies (CP) and four Patent and Proprietary Medicine Stores (PPMS) in a specific Nigerian community.  **Limitations:**  - The sample size is limited, because of the time and cost-effectiveness of the study.  - The study cannot be generalized to the whole of Nigeria. |
| 18 | [51] | Therapeutic advances in drug safety | - Adverse drug reaction (ADR)  - mHealth (mobile phone caller tunes) | - Qualitative study | - Participants indicated a lack of information about reporting ADRs as well as a desire to employ mobile phone caller tunes to encourage patients reporting of ADRs.  - There is a need for the creation and testing of caller tunes patients reporting of ADRs. | **Strengths:**  - The study examined the significance of mHealth technology for the promotion of reporting ADRs.  **Limitations:**  - Factors that could impact the production and usage of caller tunes to transmit medication safety information were not examined in the study. |
| 19 | [52] | Malaria journal | - Uncomplicated malaria  - mHealth | - Prospective observational cohort study | According to the findings of the study, telephone follow-up should be explored for monitoring pharmacological adverse events in low-resource areas. | **Strengths:**  - A very large sample size was used in the study and strong quantitative analysis was performed to analyse the data collected.  **Limitations:**  - The study may not be carried out without funding from an external institution. The cost implication of telephone follow-up and home-visit is high. |
| 20 | [53] | Frontiers in global women's health | - Maternal and child health service  - Digital technology (video training, data digitization) | - Qualitative study  - Semi-structured interview | Concurrent and persistent implementation of video training and data digitization at scale allowed by SatCom and 3G mobile services are viable options for promoting changes in personnel motivation and trust as well as reported maternal child health care service. | **Strengths:**  - The research was carried out in remote areas of Nigeria (three different states) to enhance maternal and child healthcare delivery.  **Limitations:**  - The scope of the study is only applicable to a low-resource rural area. Meanwhile, urban settings may not be aware of the implementation of digital health intervention. |
| 21 | [54] | Telemedicine journal and e-health: the official journal of the American Telemedicine Association | - Birth Outcomes  - Mobile telephone follow up | - Observational Cohort study | - The study found that mobile telephone communications have made an invaluable intervention in monitoring birth outcomes. | **Strengths:**  - The authors observed pregnant women from their first antenatal visits and recorded the potential adverse birth outcomes throughout the pregnancy.  - Therefore, regardless of whether the pregnant woman was delivered in/outside the hospital, the author used a mobile telephone follow-up to monitor stillbirths and neonatal deaths.  **Limitations:**  - The outcome of observational study is somewhat unreliable than resolving to empirical evidence. |
| 22 | [55] | JMIR mHealth and uHealth | - Voluntary blood donation  - Mobile phone caller tunes | - Cross-sectional study  -Questionnaire survey | The findings give empirical support for developing caller tunes to encourage blood donation in Ghana. According to the study, making caller tunes free is especially important for nonblood donors who do not have caller tunes. | **Strengths:**  - The study used a very strong statistical method – structural equation modelling – to test for the hypothesis  **Limitations:**  - The study was conducted in a particular hospital in Ghana, hence generalizing both rural and urban settings, not to talk of Sub-Saharan Africa, is out of context. |
| 23 | [56] | Exploratory research in clinical and social pharmacy, | - Medical adherence  - Mobile caller tunes (Application of technology acceptance model (ATM)) | - Cross-sectional study | - In terms of simplicity and use, those who perceive ATM to improve medicine adherence were higher among those who have good intentions in viewing the application.  - Availability as a free download was also connected with greater favourable attitudes among individuals who previously used caller tunes. | **Strengths:**  - The study is strong in the area of developing the ATM to improve medicine adherence in Ghana.  **Limitations:**  - The study does not consider the possibility of moderators in the study outcomes. These include but are not limited to participants’ age, whether they are currently using caller tunes, and their thoughts about the ease of using caller tunes. |
| 24 | [57] | Frontiers in global women's health, | - Extension of Maternal Health Service (MHS) to rural and distant areas  - eHealth  - Satcom, 3G mobile networks | - Cross-sectional study | Through improved data handling for healthcare decisions, the level of maternity care delivery, increased attendance at health facilities, and service consumption, digital technology can have long-term effects on health professionals, patients, and the healthcare systems. | **Strengths:**  - For the period of two years (2017 – 2019), the study distributed video training and data digitization intervention to 62 healthcare facilities in the regions with a lack of satCom and 3G mobile networks.  **Limitations:**  - The study mentioned southwest Nigeria, but it only covered health facilities in the rural segment in one state among the 5 Southwestern states.  -The study does not investigate how government can intervene to improve eHealth applications in rural areas. |
| 25 | [58] | JMIR mHealth and uHealth | - Training primary healthcare workers  - eHealth (Video Training | - Mixed methods  - Uncontrolled Before and After the study | This study discovered that video training backed by eHealth technology is a practical and acceptable technique for improving clinical knowledge, attitudes, and reported practices in maternal, newborn, and child healthcare. | **Strengths:**  -The study investigated the feasibility and acceptability of eHealth tools in improving service delivery and performance of primary health workers in Nigeria.  **Limitations:**  - The study only covered three states in Nigeria. This may be due lack of funds.  - Also, there was no mention of reaching the rural areas of the covered states. |
| 26 | [59] | BMC health services research | - General healthcare service  - Telehealth | - Questionnaire survey-based design | Telehealth activities aided in the improvement of medical diagnostics in cardiology and obstetrics, as well as the on-site patient monitoring system. | **Strengths:**  - The study examined the economic benefit of telehealth in three different district hospitals in Mali  **Limitations:**  - There was hypothesis testing to improve the accuracy of the result. Only frequency and percentage do not suffice. |
| 27 | [60] | Procedia - Social and Behavioral Sciences | - Universal health coverage  - News and social media. | - Inductive content analysis | - The use of new and social media serves as an avenue to present post-administrative and personnel structure information and as well generate quick feedback from the client. | **Strengths**:  - The study indicated that the benefit of news and social media has not been properly explored. Majority of the teaching and specialist hospitals utilize it for publicity  **Limitations**:  The study proposes that Nigeria should incorporate the idea of news and social media platforms but does not explain the exact way they can use to harness their proper benefit. |
| 28 | [61]. | Yearbook of medical informatics | - General healthcare service  - ICT (telemedicine) | - Open-source research  - Experimental studies | - The RAFT project for continuing medical education in French-speaking Africa, the teleradiology project in Mali, and the "EQUI-ResHuS" project for equal access to health through ICT in Mali are all examples of ICT initiatives discussed in the study. | **Strengths:**  - The study was specific to using RAFT (Réseau en Afrique Francophone pour la Télémédecine) Network. To improve healthcare delivery in Mali  **Limitations:**  - The findings and conclusion on the use of RAFT are shallow. |
| 29 | [62] | SAGE open medicine | - Recruiting healthcare professionals in rural areas  - eHealth (ICT) | - Longitudinal study  - Questionnaire survey-based design | - The studies demonstrated that remote practitioners had high telehealth sensitivity in general.  - Attitudes toward telemedicine, observed effect on recruiting and retaining, and hurdles to retention and recruiting all played a role in explaining the reported influence of telehealth on recruitment and retention. | **Strengths:**  - The study assessed the perceived impact of telemedicine on the hiring and retention of medical professionals in Mali's rural locations.  **Limitations:**  - The study does not include the actual demonstration of telehealth in recruiting and retaining professional workers in a remote area.  - It only seeks the perception of the healthcare workers in a remote area. |
