# Supplementary figures and images for: Digital Interventions for Managing Medication and Health Care Service Delivery in West Africa: Systematic Review
Source: J Med Internet Res. 2024 Oct 9;26:e44294. doi: 10.2196/44294 (PMC11499747; doi:10.2196/44294)

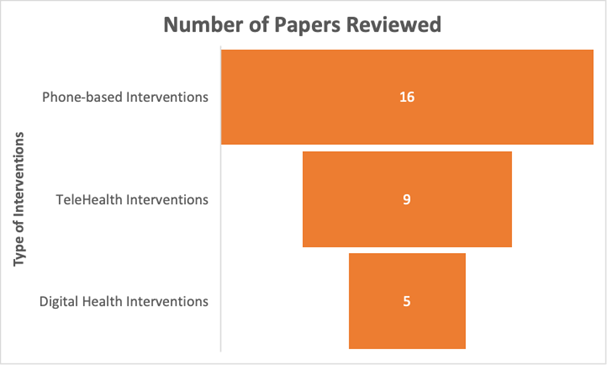

Supplement: Multimedia Appendix 3 [file jmir_v26i1e44294_app3.png]
